# Real-World Outcomes and Trends From a Prospective Japanese Transcatheter Aortic Valve Replacement Registry

**DOI:** 10.1016/j.shj.2026.100854

**Published:** 2026-05-08

**Authors:** Santiago Garcia

Postmarket approval studies, whether compulsory or voluntary, are designed to provide “reasonable assurance” that medical devices are safe and effective in clinical practice.^1^ In the United States, the STS/ACC TVT (Society of Thoracic Surgeons/American College of Cardiology Transcatheter Valve Therapy) Registry has provided important benchmarking data regarding the safety and real-world outcomes of transcatheter aortic valve replacement (TAVR), as the technology expanded from inoperable to lower-risk aortic stenosis patients.^2^ Similar initiatives have been replicated in other countries.^3^, ^4^, ^5^

In this issue of the journal,^6^ Hioki et al. present 10-year data (2013–2023) from 18 TAVR centers in Japan. The authors used the OCEAN-TAVI (Optimized transCathEter vAlvular interveNtion transcatheter aortic valve implantation) registry, a multicenter, prospective, observational study that includes patients undergoing TAVR in Japan to evaluate temporal trends in patient characteristics and short-term outcomes over the last decade. After excluding valve-in-valve procedures and patients with end-stage renal disease on hemodialysis, a total of 13,245 patients were included in the analysis.

The study contains several notable findings that expand from^1^ *who* is getting TAVR,^2^ *how* the therapy is being applied, and^3^ *what* trends we are seeing in valve utilization and relevant clinical outcomes.

First, despite indication expansion to lower-risk patients, TAVR continues to be applied to elderly patients in Japan with a median age of 85 (81–88) that did not change significantly throughout the study period. This should not be equated with lack of progress. Indication expansion is occurring in Japan—at the beginning of the study period, 1 in 5 patients receiving TAVR was low risk, whereas the proportion of low-risk patients was 1 in 3 at the end of the study period. However, indication expansion is certainly occurring at a slower pace than in Western societies. This may reflect country-specific reimbursement policies and cultural norms rather than gaps in knowledge or evidence. Second, unlike Western TAVR registries, which tend to have more males than females, OCEAN-TAVI is predominantly (67%) female patients; this has implications for device sizing, as there is a higher prevalence of small aortic annulus in women.^7^ Similarly, the study population had a lower body surface area (1.3–1.5 m^2^) than Western populations, which may favorably influence the risk of patient–prosthesis mismatch. Second, several trends in procedural characteristics suggest a shift toward “minimalistic” approaches, including increased adoption of local anesthesia/conscious sedation and transfemoral access. These trends are similar to United States and European registries.^2^, ^3^, ^4^, ^5^ There has also been increased adoption of self-expanding valves, rising from approximately 20% of TAVR procedures at the beginning of the study period to nearly 50% by the end. Notably, this shift has occurred without an increase in pacemaker or ≥ moderate paravalvular regurgitation rates. In fact, during the study period, there has been a significant decrease (8% relative risk per year) in pacemaker rates, which have remained in single digits (around 5%–6%) throughout.

Finally, short-term procedural outcomes have remained stable during the study period, with 30-day mortality around 1% and stroke rates of 2%–3%. The results should be interpreted in the context of the study population’s age (median age, 85; 81–88) and surgical risk (median STS, 5.6%). Given these considerations, the totality of the evidence suggests that the “real-world” clinical application of TAVR in Japan has been extremely safe and effective.

Certain limitations of the registry, including lack of long-term data, quality of life measures, and site-specific metrics (e.g., procedural volumes), should be acknowledged.

Country-specific registries are important not only because they shed light on real-world outcomes and practices but also because they provide essential benchmarking information for policymakers, health care providers, and patients. Cross-country comparisons are always fraught with risks and are beyond the scope of this manuscript. Nonetheless, this type of analysis, in conjunction with other TAVR registries, indicates that the commercial adoption of TAVR around the world has met or exceeded safety expectations and could provide a template for other transcatheter technologies, including mitral and tricuspid replacement.

## Funding

The authors have no funding to report.

## Disclosure Statement

S. Garcia is a consultant for Edwards Lifesciences, Medtronic, Abbott Vascular, Boston Scientific, Anteris, Capstan Medical, and B. Braun; is a proctor for Edwards Lifesciences and Abbott Vascular; is an advisory board member for Medtronic and Boston Scientific; and has stock options in Capstan Medical.
